# First validated liquid chromatography–tandem mass spectrometry method for simultaneous quantification of propranolol and 4-hydroxypropranolol in pig plasma and dried blood spots and its application to a pharmacokinetic study

**DOI:** 10.14202/vetworld.2026.15-28

**Published:** 2026-01-06

**Authors:** Anisa Bardhi, Domenico Ventrella, Alberto Elmi, Ronette Gehring, Davide Martelli, Ilaria Troisio, Maria Laura Bacci, Andrea Barbarossa

**Affiliations:** 1 Department of Veterinary Medical Sciences, University of Bologna, Ozzano Emilia (BO), Italy; 2 Health Sciences and Technologies-Interdepartmental Centre for Industrial Research (CIRI-SDV) - University of Bologna, Ozzano dell’Emilia (BO), Italy; 3 Department of Veterinary Sciences, University of Pisa, Pisa, Italy; 4 Department of Population Health Sciences, Institute for Risk Assessment Sciences (IRAS), Faculty of Veterinary Medicine, Utrecht University, Utrecht, the Netherlands; 5 Department of Biomedical and Neuromotor Sciences, University of Bologna, Bologna, Italy

**Keywords:** 4-hydroxypropranolol, beta-blocker, dried blood spots, liquid chromatography–tandem mass spectrometry, microsampling, pharmacokinetics, pigs, propranolol

## Abstract

**Background and Aim:**

Propranolol is a widely used non-selective beta-adrenergic blocker in human medicine, with well-characterized pharmacokinetics (PK) in humans but virtually no data available for pigs, a species of growing biomedical relevance. Furthermore, no validated bioanalytical methods exist for propranolol or its primary metabolite, 4-hydroxy-propranolol, in porcine matrices. This study aimed to develop and validate a rapid, sensitive, and reliable liquid chromatography–tandem mass spectrometry (LC–MS/MS) method for the simultaneous quantification of propranolol and 4-hydroxypropranolol in pig plasma and dried blood spots (DBS), and to apply it in a preliminary PK investigation in pigs.

**Materials and Methods:**

Sample preparation involved simple protein precipitation (plasma) or solvent extraction (DBS) using acetonitrile–water mixtures, followed by chromatographic separation on a Bridged ethyl hybrid C18 column (50 × 2.1 mm, 1.7 μm; 4-min run). Detection was performed in Multiple reaction monitoring mode with propranolol-d7 as the internal standard. Validation followed EMA ICH M10 guidelines, assessing linearity, accuracy, precision, matrix effects, recovery, and stability. The method was then applied to plasma samples from five juvenile female pigs receiving oral propranolol (3 mg/kg, q8 h).

**Results:**

The method demonstrated excellent linearity (r^2^ > 0.99) and acceptable accuracy and precision (±15%) across 2–500 ng/mL (propranolol) and 1–400 ng/mL (4-hydroxypropranolol). Recoveries ranged from 83% to 116% (plasma) and 81%–113% (DBS), with no matrix interference or carry-over. In vivo PK data revealed rapid absorption (Tmax 1.14 ± 0.63 h), moderate elimination (t½ 2.19 ± 0.86 h), and a mean Cmax of 112.02 ± 81.87 ng/mL. Notably, 4-hydroxypropranolol was undetectable in all plasma samples, suggesting species-specific metabolic differences.

**Conclusion:**

This study reports the first validated LC–MS/MS assay for propranolol and 4-hydroxypropranolol in pigs and demonstrates its successful application in a PK study. The method’s simplicity, short runtime, and compatibility with DBS microsampling make it ideal for preclinical and veterinary research, minimizing animal stress and sampling volume. Absence of 4-hydroxypropranolol highlights interspecies metabolic variability and warrants further investigation into propranolol biotransformation pathways in swine and other translational models.

## INTRODUCTION

**Figure 1:**
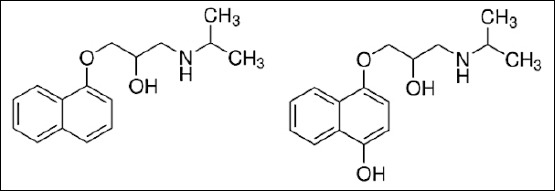
Molecular structures of propranolol and 4-hydroxypropranol.

Propranolol is a non-selective beta-adrenergic blocker that antagonizes the effects of catecholamines, such as adrenaline and noradrenaline, at both β_1_- and β_2_-adrenergic receptors, thereby suppressing sympathetic-mediated cardiovascular responses [1, 2]. This highly lipophilic compound can be administered either intravenously or orally, exhibiting complete absorption across routes. In humans, propranolol undergoes extensive hepatic metabolism, with only about 25% of the administered dose reaching systemic circulation and a plasma half-life of 3–6 h [3]. The cytochrome P450 enzyme CYP2D6 is primarily responsible for its biotransformation, converting propranolol to 4-hydroxypropranolol (4-OH-propranolol), its major active metabolite [4]. This metabolite reaches peak concentrations comparable to propranolol but displays a markedly shorter half-life [5, 6]. Figure 1 illustrates the molecular structures of propranolol and 4-OH-propranolol.

Clinically, propranolol is widely prescribed in adults for the management of hypertension, angina pectoris, and cardiac arrhythmias, as well as for secondary prevention of myocardial infarction [3, 7, 8]. In pediatric medicine, it is used for both congenital and acquired cardiovascular disorders and as the first-line therapy for infantile hemangiomas [4, 9–15]. Beyond its cardiovascular effects, propranolol is employed in several non-cardiac indications, including migraine prophylaxis, essential tremor, anxiety, hyperthyroidism, and pheochromocytoma [16, 17].

Recent studies have explored the immunomodulatory potential of propranolol, particularly its ability to influence the autonomic regulation of immune and inflammatory pathways. In animal models, sympathetic activation through the splanchnic nerves has been shown to modulate innate immune responses to bacterial infections [18, 19], suggesting that pharmacological blockade with propranolol could serve as a less invasive alternative to surgical denervation [20, 21].

From a translational perspective, pigs constitute a valuable large-animal model due to their close similarity to humans in cardiovascular anatomy, physiology, and drug metabolism. Consequently, swine are increasingly utilized in comparative pharmacology as a bridge between preclinical and clinical research, offering an ethically acceptable alternative to non-human primates within a One Health framework [22, 23]. Despite this significance, existing validated liquid chromatography–tandem mass spectrometry (LC–MS/MS) methods for propranolol quantification have been developed exclusively in humans [4, 24–30], and no validated analytical approaches are currently available for pig plasma or dried blood spots (DBS).

This study aimed to develop, validate, and apply a sensitive and reliable LC–MS/MS method for the simultaneous quantification of propranolol and its major metabolite, 4-hydroxypropranolol, in pig plasma and DBS. The specific objectives were to:

1. Optimize chromatographic and mass spectrometric conditions to achieve rapid analysis, high selectivity, and minimal matrix interference.
2. Validate the method in accordance with EMA ICH M10 guidelines, assessing linearity, accuracy, precision, matrix effect (ME), recovery, stability, and carry-over in both matrices.
3. Apply the validated assay to a preliminary PK study following oral administration of propranolol (3 mg/kg) in pigs, to characterize plasma concentration–time profiles and investigate the presence or absence of 4-hydroxypropranolol as a potential species-specific metabolic marker.

The findings were expected to provide a robust analytical foundation for future PK, toxicokinetic, and translational investigations in large-animal models, supporting the refinement of experimental protocols and the broader application of DBS microsampling techniques in veterinary pharmacology.

## MATERIALS AND METHODS

### Ethical approval

All animal procedures were reviewed and approved by the Italian Ministry of Health under Legislative Decree 26/2014 (authorization/approval No. 594/2021-PR) and conducted in accordance with EU Directive 2010/63/EU and applicable national and local animal-welfare regulations. Animals were managed and experiments were reported in compliance with the Animal Research: Reporting of *In Vivo* Experiments 2.0 guidelines.

The work followed the principles of Replacement, Reduction, and Refinement (3Rs). Specifically, bioanalytical method development used drug-free plasma and blood obtained from pigs enrolled in the same approved protocol (opportunistic sampling), thereby reducing additional animal use. The pharmacokinetic application was performed in five juvenile female pigs, selected to reduce biological variability while minimizing the number of animals used.

Animals were housed in the experimental facility of the University of Bologna under controlled environmental conditions with ad libitum access to water, a standard diet, and environmental enrichment. Routine welfare monitoring was performed in accordance with European and local standards. To enable repeated blood sampling while minimizing repeated venipuncture-related stress, pigs were fitted with a central venous catheter (CVC) placed in the left jugular vein under general anesthesia at least 3 days before dosing.

Throughout the study, animals were monitored for clinical status and procedure-related discomfort. Any animal showing signs of pain, distress, or complications was to be treated according to the facility’s veterinary care procedures and, if necessary, removed from the study and managed in accordance with predefined humane endpoints.

### Study period and location

The study was conducted between May 2022 and November 2024 at the Porcine Experimental Facility and the Laboratory of Pharmacology, Toxicology, and Food Chemistry within the Department of Veterinary Medical Sciences of the University of Bologna, located in Ozzano dell’Emilia.

### Pre-experimental sample collection

Before the start of the study, drug-free plasma and blood samples were collected from healthy pigs enrolled in the same experimental protocol approved according to the legislative decree 26/2014, either as controls or before propranolol administration, and made available to the LC–MS/MS laboratory for method development.

### Study design

This study consisted of a method development and validation phase, followed by a PK application in pigs.

### Animals, housing, and welfare

Five juvenile (4 months old) conventional hybrid female pigs (mean body weight 43 kg at the start of the study) were used in this study. To reduce variability due to sex-related physiological differences, only females were included. At weaning, the animals were purchased from a local conventional breeding farm (Suimax, 018BO073) and transported to the experimental facility of the Department of Veterinary Medical Sciences at the University of Bologna. For the duration of the trial, pigs were single-housed in standard pens, fed a standard pellet diet (Big 30–80, CESAC s.c.a., Italy) twice a day, with ad libitum access to water, and maintained under a 12:12 h light/dark cycle (minimum 50 lux during light periods) at 22°C ± 1°C. Environmental enrichment and regular welfare monitoring were performed in accordance with European and local guidelines. To minimize stress, pens were equipped with both edible and chewable environmental enrichments. The microbiological status of the facility is officially Pseudorabies-free and Swine Vesicular disease-free, and animals tested negative for porcine reproductive and respiratory syndrome, porcine parvovirus (PPV), and porcine circovirus (PCV). At least 3 days before propranolol administration, animals with a mean body weight of 43 kg were equipped with a CVC inserted in the left jugular vein under general anesthesia, allowing for repeated blood sampling.

### Drugs and reagents

Analytical standards of propranolol hydrochloride (purity 98%; catalog number: P840013), 4-hydroxypropranolol (purity 98%; catalog number: H952545), and propranolol-d7 (purity 98%; catalog number: P831803) were purchased from Toronto Research Chemicals (Toronto, Canada). Acetonitrile, methanol (MeOH), and formic acid (all LC–MS grade) were obtained from Merck (Milano, Italy). Freshly produced ultrapure water (Millipore, Milano, Italy). Whatman 903 Protein Saver Cards (Whatman, UK) were used for spotting blood samples. Propranolol capsules (immediate-release galenic formulation) were administered using a food vehicle such as yogurt to enhance palatability and facilitate oral intake while allowing precise dose adjustment according to each animal’s body weight.

### Preparation of the stock and working solutions

Individual stock solutions of propranolol, 4-hydroxypropranolol (4-OH-propranolol), and propranolol-d7 (used as internal standard, IS) were prepared by accurately weighing the appropriate amount of each compound in its pure powdered form using an analytical balance and dissolving it in MeOH using volumetric flasks. The preparation details are summarized in Table 1. Working standard solutions of propranolol and 4-OH-propranolol were prepared daily by serial dilution of the respective stock solutions with acetonitrile to obtain a range of concentrations suitable for the calibration curve and quality control (QC) samples. In particular, the working solutions had concentrations of 20, 50, 200, 500, 2,000, and 5,000 ng/mL for propranolol and 10, 40, 100, 400, 1,000, and 4,000 ng/mL for 4-OH-propranolol. All stock solutions were stored at −20°C ± 2°C in the dark, and the stability of the three compounds was assessed over 12 months of storage.

**Table 1:** Preparation and storage of stock solutions.

Compound	Amount weighed (mg)	Final volume (mL)	Stock concentration (ng/mL)	Solvent	Storage conditions
Propranolol	10	10	1,000	MeOH	−20°C
4-OH-Propranolol	5	10	500	MeOH	−20°C
Propranolol-d7	2.5	5	500	MeOH	−20°C

MeOH = Methanol

### Calibrators and QC samples

Calibrators and QC samples were prepared by adding 10 μL of appropriate working solutions to 100 μL of blank pig plasma or whole blood (hematocrit = 41%) and treated following the procedure described in the sample preparation section. For DBS samples, the cards were allowed to dry for at least 3 h at room temperature (20°C ± 2°C) before processing.

### Sample preparation for LC–MS/MS

#### Plasma samples

Plasma samples, previously thawed at room temperature (20°C ± 2°C), were prepared by transferring 100 μL of plasma and 10 μL of internal standard working solution (IS: Propranolol-d7 at 100 ng/mL in acetonitrile) into a 0.5 mL Eppendorf microtube. Protein precipitation was performed by adding 200 μL of acetonitrile, vortexing for 30 s, and centrifuging at 21,000 × *g* for 10 min at 20°C. Finally, 100 μL of the supernatant was transferred into an LC glass vial containing 200 μL of 0.1% formic acid in ultrapure water.

#### DBS samples

For the extraction of DBS, the entire spot (20 μL) was cut from the Whatman cards and transferred into 1.5 mL Eppendorf microtubes containing 400 μL of a 30:70 solution of water: Acetonitrile (v/v) and 10 μL of IS. The samples were vortexed for 1 min and then sonicated in an ultrasonic bath for 30 min. After centrifugation (10 min, 21,000 × *g*, 20°C), 100 μL of the supernatant was transferred to an LC vial containing 200 μL of 0.1% formic acid in ultrapure water.

### LC–MS/MS analysis conditions

#### UHPLC conditions

Chromatographic separation was performed using a Waters Acquity UPLC® system (Waters, Milford, MA, USA) equipped with a binary pump, thermostated autosampler, and column oven. Analytes were separated using a Waters Acquity Bridged ethyl hybrid (BEH) C18 (50 × 2.1 mm, 1.7 μm) column coupled with the relative VanGuard pre-column (Waters, Milford, MA, USA) and maintained at 40°C. A gradient program with 0.1% formic acid in water (A) and acetonitrile (B) was applied at a flow rate of 0.3 mL/min: 80:20 (VA:VB) to 5:95 in 0.75 min, held for 1.50 min, and then returned to 80:20 over 0.50 min, followed by 1.00 min of column re-equilibration (total run time 4.0 min). Samples were stored at 20°C in the autosampler, and 5 μL from each vial was injected.

#### Mass spectrometry conditions

Detection was performed using a Waters XEVO TQ-S Micro triple quadrupole mass spectrometer (Waters, Milford, MA, USA) equipped with an electrospray ionization source and operating in Multiple reaction monitoring (MRM) mode. The capillary voltage was set at +2.80 kV, and the source and desolvation temperatures were 150°C and 600°C, respectively. The cone gas was set to 50 L/h, and the desolvation gas to 900 L/h; argon was used as the collision gas. The analyte-dependent MS/MS parameters were optimized by infusing the standard solution of each analyte and the LC mobile-phase into the mass spectrometer.

#### MRM transitions and software

Table 2 shows the most abundant transitions identified for propranolol, 4-hydroxypropranolol, and propranolol-d7, along with their relative cone voltage and collision energy values. MassLynx 4.2 software (Waters, Milford, MA, USA) was used for data acquisition and analysis.

**Table 2:** Selected mass transitions (in italics, the product ions used for quantification) for propranolol, 4-hydroxypropranolol, and propranolol-d7, along with their cone voltage and collision energy optimized values.

Analyte	MRM transition (*m/z*)	Cone voltage (V)	Collision energy (eV)
Propranolol	260.2 > 116.1	15	18
	260.2 > 183.0	15	20
4-hydroxypropranolol	276.1 > 116.1	10	18
	276.1 > 173.2	10	16
Propranolol-d7	267.2 > 116.3	18	18

MRM = Multiple Reaction Monitoring.

### Method validation

The technique was validated for each analyte following the European Medicines Agency ICH M10 guideline on bioanalytical method validation and study sample analysis [31]. Validation was performed across three separate testing days for both plasma and DBS. The parameters considered included selectivity, calibration range, lower limit of quantification (LLOQ), accuracy, precision (coefficient of variation, CV%), ME, carry-over, stability, and reinjection reproducibility.

#### Selectivity and specificity

The retention times of propranolol, 4-OH-propranolol, and propranolol-d7 were determined by injecting individual pure solutions at 100 ng/mL after optimizing the chromatographic conditions. Selectivity was assessed by analyzing six blank pig plasma and DBS samples to verify the absence of interfering signals at the target compound retention times.

#### Calibration curve and LLOQ values

In each session, matrix-matched calibration curves were freshly prepared following the procedure described in the sample preparation section, including a blank sample, a zero sample (blank spiked with IS), and six calibrator levels. The calibration range (LLOQ-ULOQ) was 2.0–500.0 ng/mL for propranolol and 1.0–400.0 ng/mL for 4-OH-propranolol in both matrices. Peak area ratios between each analyte and the IS were plotted against their concentration, and a linear least square regression model was applied. Table 3 shows the calibration ranges for propranolol and 4-hydroxypropranolol in plasma and DBS. The LLOQ was defined as the lowest concentration measured in the samples that could be detected with a signal-to-noise ratio ≥10 and acceptable accuracy (within ±20%) and precision (CV <20%) after the injection of four replicates. All calibration standards should be within ±15% of the expected concentration.

**Table 3:** Calibrators, LLOQ, and QC samples prepared for plasma and DBS analysis of propranolol and 4-hydroxypropranolol.

Level	Propranolol (ng/mL)	4-hydroxypropranolol (ng/mL)
1	2.0	1.0
2	5.0	4.0
3	20.0	10.0
4	50.0	40.0
5	200.0	100.0
6	500.0	400.0

LLOQ = Lower limit of quantification, QC = Quality control, DBS = Dried blood spots.

#### Accuracy and precision

To evaluate the method’s intra- and inter-day accuracy and precision, QC samples at four different concentrations were prepared in five replicates alongside each calibration curve. Accuracy, expressed as the relative difference between the measured value and expected concentration, was evaluated at each QC level (lower limit of quantification: LLOQ; low QC: LQC; medium QC: MQC; and high QC: HQC), and considered acceptable if within ±15% of the nominal concentration. Similarly, precision, defined as the CV% among repeated individual measures, had to be <15% for each QC level.

#### ME

Potential ME was assessed using the post-column infusion technique: Blank matrix samples were injected, and 0.5 μg/mL standard solutions were co-infused at the MS interface to evaluate signal stability. The ionization suppression or enhancement effect was calculated as follows: ME % = (Response post-extracted sample/Response non-extracted neat sample − 1) × 100. Negative values indicate suppression of ionization, whereas positive values suggest enhanced ionization.

#### Recovery

The recovery of the two analytes in both matrices was evaluated by spiking two series of samples at LQC and HQC levels. One series was spiked before extraction (n = 3) and the other was spiked after extraction (n = 3). Then, the mean measured values were compared.

#### Carry-over assessment

The absence of carry-over contamination was evaluated by analyzing six drug-free plasma or DBS samples after the highest calibrator injection. The analytical response in the blank samples must be <20% of the LLOQ.

#### Stability tests and reproducibility of reinjection

Different tests were performed to assess the stability of the target analytes in the plasma and processed samples. The long-term stability of each analyte in plasma stored at −80°C was evaluated by preparing additional QCs (lowest and highest levels, n = 3) for analysis after 1, 6, and 12 months of storage. The stability of the processed samples was first investigated by reinjecting the lowest and highest QCs (n = 5) from the 1st day of validation after they had been left in the autosampler (20°C) for 24 h. For all these stability tests, the mean concentration at each condition had to be within ±15% of the nominal value. No stability tests have been conducted on DBS.

### PK study in pigs

#### Drug administration protocol

At least 3 days before propranolol administration, animals with a mean body weight of 43 kg were equipped with a CVC inserted into the left jugular vein under general anesthesia, allowing repeated blood sampling. Propranolol capsules were administered orally with biscuits (digestive, McVitie’s, UK) and/or plain white yogurt (Pascoli Italiani, IT), 3 times a day (q8h) at a dosage of 3 mg/kg, three times daily (q8h) using biscuits and/or yogurt as a vehicle to ensure palatability. Due to the nature of the study, which was preliminary and based on opportunistic sampling, no power analysis was conducted to determine the sample size.

#### Blood collection and plasma processing

Blood samples were collected on days 1, 8, and 15 at −10, 10, 20, 40, 60, 120, 240, and 410 min relative to dosing. Samples were collected into K3 EDTA tubes (S-Monovette®, Sarstedt AG and Co. KG, DE) and centrifuged for 15 min at 2500 × *g* at 4°C (Megafuge ST1R Plus-MD, Thermo Scientific, MA, US). Plasma was aliquoted and immediately stored at −80°C until LC–MS/MS analysis. DBS sampling was not performed *in vivo*; instead, the procedures were validated *ex vivo* in the laboratory using collected blank blood samples.

#### PK analysis

The PK analysis was performed using Phoenix WinNonlin 8.5 (Certara, Princeton, NJ, USA). Non-compartmental analysis was applied to plasma concentration–time data. The following PK parameters were calculated for propranolol: maximum plasma concentration (Cmax), time to reach Cmax (Tmax), elimination half-life (T1/2), area under the concentration–time curve from time 0 to the last measurable concentration and to infinity (area under the curve [AUC]0–t and AUC0–∞), apparent clearance (CL/F), volume of distribution (Vd), and mean residence time, with data reported as mean ± standard deviation (n = 5). The AUC was calculated using the linear-log trapezoidal rule. The terminal slope (λz) was determined by applying log-linear regression to the terminal portion of the concentration–time data, with a weighting scheme of 1/(Y^2^). The PK parameters were calculated using standard equations. CL was calculated as the ratio of the administered dose to the AUC (CL = Dose/AUC), and Vd was derived from the terminal phase as Vd = CL/λz [30].

## RESULTS

### Method development and validation

**Figure 2:**
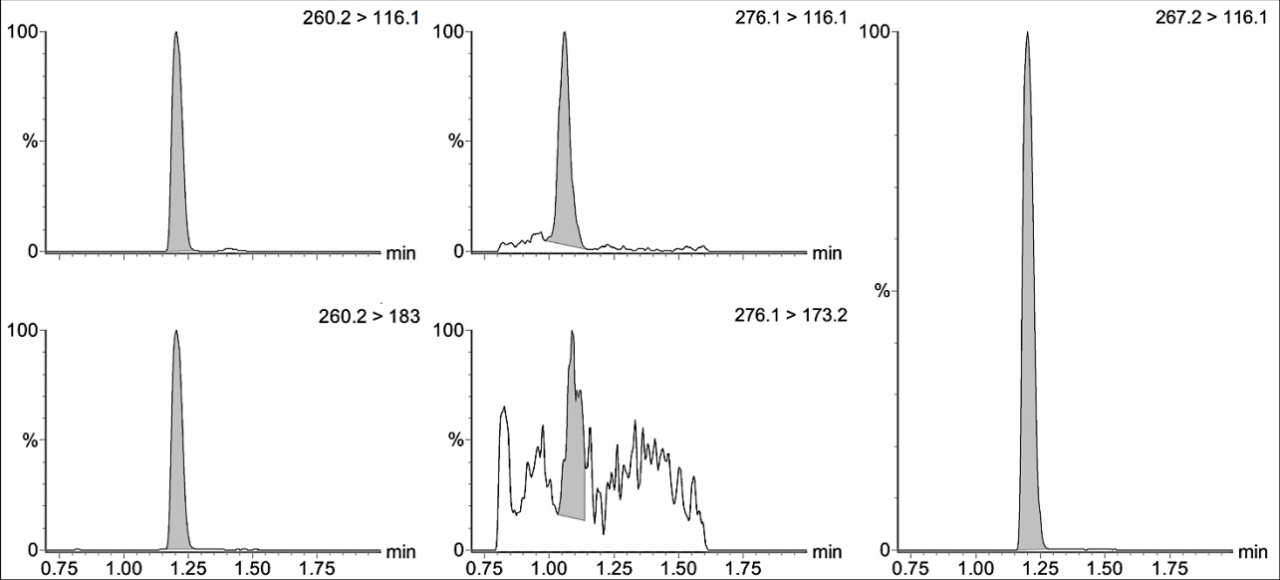
Chromatograms obtained from the Multipe Reaction Monitoring analysis of one of the plasma samples collected from pigs, including the ion transitions used for propranolol quantification (Left), 4-hydroxypropranolol (Center), and the ion transition monitored for the internal standard d7-propranolol (Right).

The retention times were 1.19 min for propranolol and propranolol-d7 and 1.07 min for 4-hydroxypropranolol (Figure 2). The absence of interfering peaks at the retention times of the target analytes after injection of blank matrix samples confirmed the specificity of the method.

The LLOQ was 2 ng/mL for propranolol and 1 ng/mL for 4-OH-propranolol in both plasma and DBS matrices. Calibration curves prepared on three independent testing days consistently exhibited a coefficient of determination (r² ≥ 0.99). Furthermore, all calibrators were within ±15% of the expected value, confirming the method’s excellent linearity across the validated concentration ranges.

Accuracy and precision at all QC levels for each matrix, under both intra-day and inter-day conditions, are presented in Table 4 (plasma) and Table 5 (DBS). No ionization suppression or enhancement was observed in the monitored transitions around the analyte retention times during the post-column infusion test, confirming the absence of ME.

**Table 4:** Intra-day and inter-day accuracy and precision in plasma analysis.

Day (n = replicates)	Propranolol		4-hydroxypropranolol	
	
	Accuracy (%)	Precision (%)	Accuracy (%)	Precision (%)
	LLOQ (2 ng/mL)		LLOQ (1 ng/mL)	
Day 1 (n = 5)	−10.0	9.1	0	10
Day 2 (n = 5)	−7.5	7.0	0.7	1.1
Day 3 (n = 5)	−5.3	7.2	−10.0	11.1
Interday (n = 15)	−7.6	7.4	−3.1	9.1
	LQC (5 ng/mL)		LQC (4 ng/mL)	
Day 1 (n = 5)	−4.0	2.1	0	2.5
Day 2 (n = 5)	−6.0	2.1	−0.8	3.9
Day 3 (n = 5)	3.3	3.0	−1.7	5.3
Interday (n = 15)	−2.2	4.8	−0.8	3.6
	MQC (50 ng/mL)		MQC (40 ng/mL)	
Day 1 (n = 5)	0.7	1.8	1.6	2.9
Day 2 (n = 5)	0.8	0.7	3.3	5.6
Day 3 (n = 5)	−1.7	5.7	2.7	6.3
Interday (n = 15)	−0.1	3.2	2.5	4.5
	HQC (500 ng/mL)		HQC (400 ng/mL)	
Day 1 (n = 5)	2.8	4.3	−0.2	0.3
Day 2 (n = 5)	3.1	1.8	0.7	1.6
Day 3 (n = 5)	−0.4	1.9	0.8	1.5
Interday (n = 15)	1.8	3.0	0.4	1.2

LLOQ = Lower Limit of Quantification, LQC = Low Quality Control, MQC = Medium Quality Control, HQC = High Quality Control

**Table 5:** Intra- and inter-day accuracy and precision in the DBS samples.

Day (n = replicates)	Propranolol		4-hydroxypropranolol	
	
	Accuracy (%)	Precision (%)	Accuracy (%)	Precision (%)
	LLOQ (2 ng/mL)		LLOQ (1 ng/mL)	
Day 1 (n = 5)	−1.1	12.1	−2.4	10.8
Day 2 (n = 5)	−1.0	12.1	6.0	7.8
Day 3 (n = 5)	3.9	8.0	2.4	10.2
Interday (n = 15)	0.6	10.3	2.0	9.5
	LQC (5 ng/mL)		LQC (4 ng/mL)	
Day 1 (n = 5)	−0.4	3.9	3.6	11.2
Day 2 (n = 5)	0	3.2	1.8	11.0
Day 3 (n = 5)	−1,4	4.8	2.6	9.9
Interday (n = 15)	−0.6	3.7	2.7	10.0
	MQC (50 ng/mL)		MQC (40 ng/mL)	
Day 1 (n = 5)	2.2	2.9	1.8	4.5
Day 2 (n = 5)	2.0	4.6	3.0	4.4
Day 3 (n = 5)	0.4	1.7	6.0	8.4
Interday (n = 15)	1.5	3.1	3.6	6.0
	HQC (500 ng/mL)		HQC (400 ng/mL)	
Day 1 (n = 5)	0.1	0.3	0.4	0.6
Day 2 (n = 5)	−0.3	1.1	2.5	4.2
Day 3 (n = 5)	0.2	0.4	0.6	0.9
Interday (n = 15)	0	0.7	1.2	2.5

LLOQ= Lower Limit of Quantification, LQC = Low Quality Control, MQC = Medium Quality Control, HQC= High Quality Control

**Figure 3:**
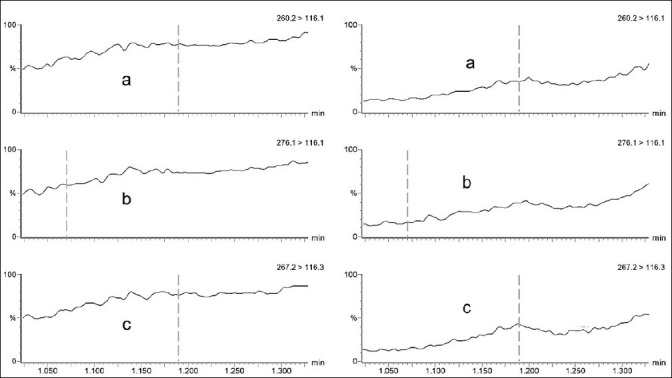
Chromatographic signals of the main mass spectrometry (MS)/MS transitions of propranolol (a) 4-hydroxypropranolol (b) 4-hydroxypropranolol, and (c) propranolol-d7 generated by the post-column infusion of standard solutions, along with the injection of a blank sample of plasma (left) and dried blood spots (right). The dashed line indicates the target analyte’s expected retention time.

Figure 3 displays the chromatographic signal obtained for the quantification transitions of the three compounds in each matrix. Comparison between samples spiked before and after extraction revealed propranolol recoveries ranging from 83% to 116% in plasma and 105%–113% in DBS, whereas 4-hydroxypropranolol recoveries ranged from 83% to 95% in plasma and 81%–107% in DBS.

The use of propranolol-d7, a deuterated internal standard, ensured reliable quantification by compensating for both systematic and random analytical variations. All drug-free samples analyzed after the highest calibrator injections produced no detectable signal, indicating the absence of carry-over contamination.

Long-term stability testing demonstrated that plasma samples stored at −80°C for 1, 6, and 12 months showed variations within ±8% compared to day 0 values. Similarly, processed samples reinjected after 24 h in the autosampler at 20°C exhibited differences within ±5%, confirming the robust stability and reproducibility of the analytical method.

### Application to a PK study

The validated LC–MS/MS method was applied to analyze plasma samples collected from a PK study investigating propranolol after oral administration of 3 mg/kg in five pigs (n = 5). The principal human metabolite, 4-hydroxypropranolol, was also examined but was not detected in any analyzed samples.

**Figure 4:**
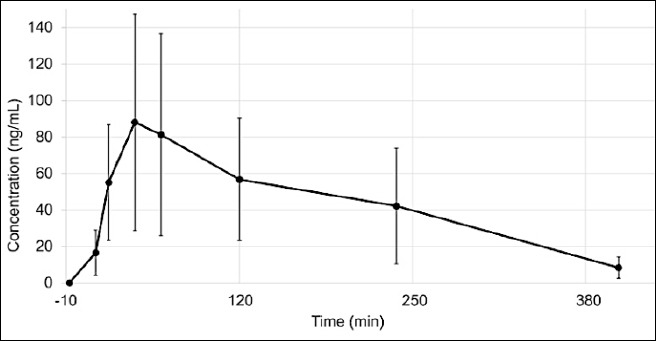
Plasma concentration–time profiles of 3 mg/kg propranolol following oral administration. Data are presented as mean ± standard deviation (SD) (ng/mL), n = 5 pigs. The error bars represent the SD of the mean.

Figure 4 illustrates the plasma concentration–time profiles of propranolol, and Table 6 summarizes the corresponding PK parameters. The mean plasma Cmax of propranolol was 112.02 ± 81.87 ng/mL, with a mean Tmax of 1.14 ± 0.63 h.

**Table 6:** Mean ± standard deviation of the pharmacokinetic parameters (noncompartmental analysis) obtained from five pigs orally administered 3 mg/kg of propranolol.

Parameter	Value
Tmax (min)	1.14 ± 0.63
Cmax (ng/mL)	112.02 ± 81.87
T1/2 (h)	2.19 ± 0.86
Vz/F (L/kg)	61.00 ± 57.29
Cl/F (L/min kg)	16.27 ± 10.61
AUC_0-∞_ (h*ng/mL)	921.59 ± 688.20
Mean residence time (MRT)_0-∞_ (h)	3.37 ± 1.16

AUC = Area under the curve

**Figure 5:**
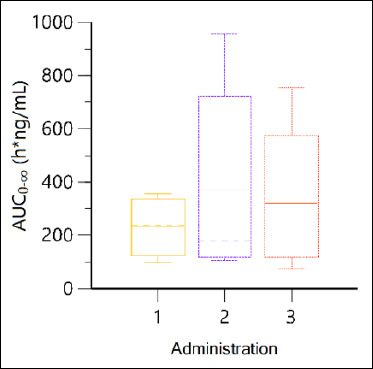
Box-and-whisker plot illustrating the variability in area under the curve_0_–∞ across the three propranolol administrations (Days 1, 8, and 15). The box represents the interquartile range, the line inside the box indicates the median, and the whiskers denote the minimum and maximum values.

The area under the concentration–time curve from time 0 to infinity (AUCINF_obs), expressed in h·ng/mL, across the three administrations is represented in Figure 5 as a box-and-whisker plot.

Marked interindividual variability was observed, particularly in Cmax and AUC values. This variability was primarily due to one animal exhibiting significantly higher plasma concentrations than the others, resulting in a global AUC CV% of 74%.

## DISCUSSION

### Method development and validation

We achieved optimal resolution, peak shape, and response for propranolol, 4-OH-propranolol, and propranolol-d7 by performing chromatographic tests with different mobile-phase compositions (water with or without pH correctors, acetonitrile, and MeOH) and gradient conditions. These outcomes were obtained using a BEH C18 column under programmed chromatographic conditions and a mobile-phase consisting of water containing 0.1% formic acid and acetonitrile. The flow rate was set at 0.3 mL/min during chromatographic runs lasting only 4 min.

After testing both acetonitrile and MeOH, the plasma sample preparation procedure involved protein precipitation with acetonitrile, which yielded the best chemical noise reduction results. This procedure avoided more labor-intensive and expensive extraction techniques, such as liquid–liquid extraction [32–34] or solid-phase extraction [24, 35, 36].

In contrast to Della Bona *et al*. [30], we employed a different approach for the DBS sample preparation protocol. Specifically, the entire DBS, corresponding to 20 μL of blood, was cut to minimize potential hematocrit-related influences [37–39]. Acetonitrile was used as the extraction solvent for plasma. The cut DBS was immersed in 400 μL of a water: acetonitrile 30:70 (v/v) mixture, and 10 μL of IS solution was added. The combination of these solvents, along with ultrasonication and centrifugation, yielded immediate, satisfactory extraction results. Both sample types (plasma and DBS) were diluted threefold in an aqueous solution containing 0.1% formic acid before LC–MS/MS analysis.

All the evaluated validation parameters produced successful outcomes for both matrices, fully meeting the criteria established by the European guidelines [31]. Stability tests conducted on plasma provided evidence that the drug remains stable for at least 12 months when stored at −80°C. However, no stability tests were conducted on DBS. Nevertheless, a study conducted by Della Bona *et al*. [30] demonstrated that propranolol remains stable in DBS up to 1 month when stored at room temperature, 4°C, and −20°C.

Overall, the validated LC–MS/MS method provides a rapid, sensitive, and straightforward analytical approach, featuring a chromatographic run time of only 4 min, robust sensitivity (LLOQ 1–2 ng/mL), and minimal sample preparation through protein precipitation. This method represents a practical and reliable tool for PK studies in pigs, while avoiding costly and time-consuming extraction procedures.

### Application to a PK study

Pigs have been used as animal models to study various diseases [40, 41] and to investigate the effects of propranolol on electroencephalographic activity and the minimum alveolar concentration of isoflurane [42]. To the best of our knowledge, no studies have examined the PK of propranolol in pigs. Nonetheless, as already stated, the porcine species is currently heavily relied on for non-clinical trials in several fields where propranolol may be relevant, such as immunity, cardiovascular surgery, and oncology. Therefore, gaining more accurate knowledge of the PK profile of propranolol in pigs may help in refining experimental translational protocols.

Although the LC–MS/MS method could detect 4-OH-propranolol, the main active metabolite of propranolol in humans, it was undetectable in pig plasma. Despite its established presence in humans [4–6], the absence of this metabolite in pigs may reflect species-specific differences in hepatic metabolism, possibly due to variations in CYP2D6 or other oxidative enzymes. Because no studies in the literature address this evaluation, we can hypothesize that this metabolite is likely not present in pigs, or, in any case, is present at levels lower than 1 ng/mL. Future studies will be helpful to better investigate the presence of 4-OH-propranolol in pigs.

No data are currently available for other animal species, such as dogs and horses; therefore, it remains unclear whether this finding is unique to pigs or generalizable across non-human models. The observed variability in plasma concentrations, particularly across repeated administrations, may be partly attributed to oral dosing challenges.

Figure 5 shows the Box-and-Whisker Plot, which highlights the variability in AUC_0_–∞ across the three administrations. The second administration (day 8) exhibited the highest variability, whereas the first administration (day 1) showed the lowest variability with consistently smaller values. The third administration (day 15) fell in between. This observed variability in plasma concentrations may be partly attributed to challenges with oral dosing, as some animals either refused or incompletely ingested the drug after the first administration, resulting in fluctuations in the actual dose delivered. Future studies could explore alternative administration routes or stricter dosing control to minimize variability.

Although the DBS method was successfully validated, it was not applied *in vivo*, precluding direct comparison of PK data between plasma and DBS. Finally, the absence of 4-hydroxypropranolol in pig plasma may reflect either very low metabolite levels or species-specific metabolic differences, and further studies are needed to confirm this observation.

Several limitations should be acknowledged in this preliminary study. Only five pigs were enrolled, limiting the statistical power of the findings, and all animals were female, preventing assessment of potential sex-related PK differences. Enrolling a larger number of pigs would enable a more comprehensive investigation of variability in propranolol PK in this species. Variability in oral administration likely contributed to fluctuations in plasma concentrations, as some animals ingested only partial doses, limiting data consistency.

In this phase of the research, although the DBS method was successfully validated, it was not applied *in vivo*, precluding direct comparison of PK data between plasma and DBS. Finally, the absence of 4-hydroxypropranolol in pig plasma may reflect either metabolite levels below the LLOQ or species-specific differences in metabolism. Nonetheless, the validated analytical tools are a valuable resource for future investigations.

## CONCLUSION

This study successfully developed and validated a rapid, sensitive, and reproducible LC–MS/MS method for the simultaneous quantification of propranolol and its major human metabolite, 4-hydroxypropranolol, in both plasma and DBS matrices. The method exhibited excellent specificity, linearity (r^2^ ≥ 0.99), accuracy, and precision, with LLOQs of 2 ng/mL and 1 ng/mL for propranolol and 4-OH-propranolol, respectively. The protein precipitation extraction and short 4-min chromatographic run time offered a simple, cost-effective alternative to conventional extraction techniques, while ensuring high analyte recovery (83%–116%) and no matrix interference or carry-over. Stability tests further demonstrated that propranolol remains stable in plasma for up to 12 months at −80°C, confirming the method’s robustness and suitability for long-term PK applications.

Application of the validated method to a PK study in pigs revealed a mean Cmax of 112.02 ± 81.87 ng/mL, a Tmax of 1.14 ± 0.63 h, and a moderate elimination half-life, indicating rapid absorption and distribution following oral dosing. Notably, 4-hydroxypropranolol was undetectable in all plasma samples, suggesting potential species-specific metabolic differences compared with humans, possibly linked to variations in CYP2D6 activity. The observed interindividual variability, especially in Cmax and AUC values (CV = 74%), was primarily attributed to inconsistencies in oral intake among animals, reflecting a common challenge in voluntary dosing studies.

From a practical standpoint, this validated LC–MS/MS method provides a powerful analytical tool for PK and toxicokinetic research in large-animal models. Its simplicity, speed, and compatibility with DBS microsampling make it especially valuable for preclinical and translational studies where minimizing animal stress and sample volume is critical. The study also reinforces the translational relevance of the pig model, bridging preclinical pharmacology and human therapeutic research.

However, some limitations must be acknowledged. The small sample size (n = 5) and inclusion of only females limit statistical power and preclude assessment of sex-related PK differences. Moreover, the DBS method, though successfully validated, was not tested *in vivo*, and the absence of 4-OH-propranolol requires further biochemical confirmation.

Future research should focus on expanding sample size, including both sexes, and evaluating alternative administration routes to minimize dosing variability. Additional metabolic and enzymatic studies are also warranted to clarify propranolol’s biotransformation pathways in pigs and other non-human species. Extending the validated method to multi-analyte quantification and DBS field application could further enhance its utility in veterinary and translational pharmacology.

The developed LC–MS/MS assay offers a robust, precise, and versatile platform for propranolol quantification and PK evaluation in pigs. It establishes a foundation for future preclinical, comparative, and One-Health-oriented pharmacological investigations, advancing the reliability and ethical efficiency of animal-based biomedical research.

## DATA AVAILABILITY

Validation results have already been presented in the manuscript. Additional raw data from the PK study are available from the AMSActa Institutional Research Repository of the University of Bologna (Bardhi *et al.*, 2025: Raw Data on Propranolol in Pig Plasma).

## AUTHORS’ CONTRIBUTIONS

AB (Andrea Barbarossa): Supervision, visualization, conceptualization, methodology, validation, data curation, software, drafted and revised the manuscript. AB (Anisa Bardhi): Conducted the study, methodology, validation, formal analysis, data curation, visualization, and drafted the manuscript. DV and MLB: Conceptualization and drafted and revised the manuscript. AE, IT, and DM: Investigation and reviewed the manuscript. RG: Methodology, software, formal analysis, and reviewed the manuscript. All authors have read and approved the final version of the manuscript.
